# Characterization of the Common Genetic Basis Underlying Seed Hilum Size, Yield, and Quality Traits in Soybean

**DOI:** 10.3389/fpls.2021.610214

**Published:** 2021-02-25

**Authors:** Qingsong Zhao, Xiaolei Shi, Long Yan, Chunyan Yang, Cong Liu, Yan Feng, Mengchen Zhang, Yongqing Yang, Hong Liao

**Affiliations:** ^1^The Key Laboratory of Crop Genetics and Breeding of Hebei, Institute of Cereal and Oil Crops, Hebei Academy of Agricultural and Forestry Sciences, Shijiazhuang, China; ^2^Root Biology Center, College of Resources and Environment, Fujian Agriculture and Forestry University, Fuzhou, China

**Keywords:** seed hilum size, genetic effects, QTL, seed yield, seed quality, breeding

## Abstract

Developing high yielding cultivars with outstanding quality traits are perpetual objectives throughout crop breeding operations. Confoundingly, both of these breeding objectives typically involve working with complex quantitative traits that can be affected by genetic and environmental factors. Establishing correlations of these complex traits with more easily identifiable and highly heritable traits can simplify breeding processes. In this study, two parental soybean genotypes contrasting in seed hilum size, yield, and seed quality, as well as 175 F_9_ recombinant inbred lines (RILs) derived from these parents, were grown in 3 years. The *h^2^_*b*_* of four hilum size, two quality and two yield traits, ranged from 0.72 to 0.87. The four observed hilum size traits exhibited significant correlation (*P* < 0.05) with most of seed yield and quality traits, as indicated by correlation coefficients varying from -0.35 to 0.42, which suggests that hilum size could be considered as a proxy trait for soybean yield and quality. Interestingly, among 53 significant quantitative trait loci (QTLs) with logarithm of odds (LOD) values ranging from 2.51 to 6.69 and accounting for 6.40–16.10% of genetic variation, three loci encoding hilum size, *qSH6.2*, *qSH8*, and *qSH10*, colocated with QTLs for seed yield and quality traits, demonstrating that genes impacting seed hilum size colocalize in part with genes acting on soybean yield and quality. As a result of the breeding efforts and field observations described in this work, it is reasonable to conclude that optimizing hilum size through selection focused on a few QTLs may be useful for breeding new high yielding soybean varieties with favorable quality characteristics.

## Key message

Hilum size could be used as a proxy trait for breeding soybean cultivars with high yielding and outstanding quality in field condition.

## Introduction

Soybean (*Glycine max* L. Merr.) is one of the most widely cultivated crops worldwide, with its protein- and oil-rich beans and contributing about 69% of the protein and 30% of the oil consumed by humans and livestock (Van and McHale, 2017). Consequently, improvements in bean yield and quality traits are perennially targeted as primary objectives in soybean breeding programs across the globe. However, yield and quality traits are complex, quantitative traits that are impacted by both genetic and environmental factors (Li et al., 2008). The continuous nature of these traits and marginal variation among top performing lines can make it difficult to differentiate individual lines in field experiments. In some cases, traits with simpler genetic components, or that are more robust under field conditions, can be used as proxy traits for yield and quality traits. For instance, soybean yield has been associated with seed size and individual plant architecture (Hartung et al., 1981). In other words, soybean plants with ideal shoot architectures and suitable seed sizes are more likely to produce high yields in larger-scale production. In situations such as this, breeders successfully improved soybean yield and quality by evaluating a relatively simple set of traits that are mainly controlled by few genetic loci and/or less affected by the environment. For example, relevant soybean growth characteristics were found to be largely controlled by only two genetic loci, *Dt1* and *Dt2*, which have been proven to play critical roles in remolding plant shoot architecture and enhancing soybean field yield (Bernard, 1972; Cober and Morrison, 2010). Therefore, characterization of the genetic basis of relatively simple traits and their roles in improving soybean field yield and quality traits may facilitate soybean breeding, especially in programs incorporating marker-assisted selection (MAS).

In soybean, the hilum, which connects the pod wall with the seed coat, provides a pathway for delivering nutrients and photosynthates to the developing embryo and is, therefore, a critical tissue for seed development (Hardham, 1976; Thorne, 1981). Several reports have begun to outline just how important this connection is. For one, the major and minor hilum axes lengths have been positively correlated with protein content and individual seed weight (Barion et al., 2016). Plus, plant seeds with intact hilums exhibit relatively high seed vigor (Kumar et al., 2019), whereas plants with injured hilums produce poor quality seeds that are susceptible to significant yield losses, possibly due to bacterial infections and reduced nutrient flows (Hsieh et al., 2005). In addition, the seed hilum is also the channel for water uptake and efflux during germination (Pietrzak et al., 2002; Zhang et al., 2004; Muramatsu et al., 2008; Jaganathan et al., 2019) and as the seed matures (Hyde, 1954). Furthermore, hilum attributes might have been selected during domestication, since this tissue serves as a hygroscopically activated valve in the impermeable epidermis of the testa, which is critical for seed dormancy (Hyde, 1954). Corroborating evidence has also been produced in more invasive experiments in which exposure of the hilum to moderate doses of ionizing radiation led to genomic mutations (Arase et al., 2011) and alterations in seed growth and development (Li et al., 2011). Overall, previous studies have determined that hilum morphology and health significantly influence seed weight and quality. Unfortunately, the hilum, as a relatively simple trait, has not attracted more attention from breeders, as the currently available data do not provide useful information about the genetic basis for seed hilum roles in improving soybean yield and seed quality in field conditions. In initiating this present work, we decided that determining the genetic basis of hilum traits and exploring genetic resources available for controlling soybean seed hilum morphology might facilitate MAS breeding efforts aimed at improving soybean yield and seed quality in agricultural settings.

In this study, two soybean cultivars with contrasting phenotypes in seed yield, seed quality, and hilum traits were crossed to construct a genetic population consisting of 175 F_9__:__11_ recombinant inbred lines (RILs). The parents and their offspring were evaluated in 3 years of field experiments to determine (1) if seed hilum traits are correlated with yield and quality traits and, if so, then (2) what is the common genetic basis. In short, the objective of this study is to explore the common genetic basis underlying seed hilum size, yield, and quality traits in soybean, which possibly can be used as a proxy trait in soybean breeding program.

## Materials and Methods

### Plant Materials and Field Conditions

The two parental soybean cultivars used in this study, JD12 and NF58, contrast in hilum size, yield, and quality traits and were, therefore, employed to construct a RIL population consisting of 175 F_9_ individual plants produced through single seed descent (SSD). The tested traits of this population were evaluated in field conditions. The field trial was carried out from 2014 to 2016 at the Dishang experiment farm (E 114.48°, N 38.03°) of the Institute of Cereal and Oil Crops, Hebei Academy of Agricultural and Forestry Sciences, Shijiazhuang City, China. Soil at the experiment site belongs to the *Fluventic Ustochrept* family of soils. Basic characteristics of the top 25 cm of soil at this location were measured in 2014 as follows: pH, 8.2; organic matter, 19.3 g kg^–1^; available P (Olsen-P), 14.9 mg kg^–1^; available N, 79.4 mg kg^–1^; and available K, 161.3 mg kg^–1^. The previous crop was wheat, and the field was supplied with 900 kg/ha of compound fertilizer (N/P_2_O_5_/K_2_O = 15:15:15) as basic fertilizer and 400 kg/ha of urea as additional fertilizer during the soybean seedling elongation stage. Following local practices, no additional fertilizer was supplied during soybean growth. Irrigated water was applied one to two times according to the requirements of plants throughout development. The RILs and parental genotypes were planted in randomized complete blocks. Each parental and RIL line was grown in three replications. Twenty plants were grown in each 2 m rows spaced 0.5 m apart. This population was further used to construct a genetic linkage map to detect quantitative trait loci (QTLs) associated with hilum size as well as seed yield and quality traits.

### Plant Sampling and Measurements

When 95% of the pods have reached their mature pod color, 10 representative plants were randomly selected from the middle of each row and pooled to evaluate the average of plant seed weight (PSW) whenever seed water content was reduced to values below 11%. One hundred ten seeds were also randomly selected from each plot to measure 100 seed weight (100SW), along with seed hilum length (SHL) and seed hilum width (SHW), by manual measurements. In order to describe the relative size of the hilum, the seed hilum area (SHA) and percentage of seed hilum area in seed projected area (PSHA) were also evaluated by the following formula: SHA = SHL × SHW × π/4 and PSHA = SHA/(SL × SW × π/4). In addition, approximately 20 g of seeds were also randomly selected from each plot for evaluating protein content (PC) and oil content (OC) using a MATRIX-I (BRUKER, Germany) NIR spectrometer (Jiang et al., 2011).

### Statistical Analysis

Seed hilum, yield, and quality trait data from field trials were used for genetic analysis conducted in R using the Performance Analytics package (Peterson et al., 2018), and correlations were assessed using Pearson’s correlation coefficient and visualized using the “corrplot” package in R. Broad sense heritability (*h^2^_*b*_*) was estimated using QTL ICIMapping V4.1 (Meng et al., 2015) for each trait according to: *h^2^_*b*_* = VG/(VG + VE), with VG and VE are genetic variance and environmental variance, respectively. The Student’s *t* test was used to test for significant effects of hilum traits on yield and quality traits using SPSS19 (Gray and Kinnear, 2012).

### Genotyping by SoySNP50K Bead Chip and Construction of Genetic Linkage Map

DNA was isolated from the leaf tissue of each RIL and parent, which was then genotyped with the SoySNP50K Bead Chip as described by Song (Song et al., 2013). Alleles with single-nucleotide polymorphisms (SNPs) were called using the Genome Studio Genotyping Module v1.8.4 (Illumina, Inc. San Diego, CA). SNPs included in further analysis were those that fell into two or three discrete clusters in a SNP Graph Alt and which had both alleles present with high signal intensities (Song et al., 2013). Chi-square (χ^2^) tests were conducted for all SNPs to detect segregation distortion. SNPs with segregation distortion were removed from further analysis. After filtering, qualified SNP markers were then used to construct genetic linkage maps using IciMapping V4.1 (Meng et al., 2015) described in Yang et al. (2017).

### QTL Detection and Comparison

The multiple QTL mapping (MQM) method in MapQTL6.0 (Van Ooijen and Kyazma, 2011) was used to map QTLs associated with hilum, quality, and yield traits. The logarithm of odds (LOD) threshold was set to 2.5 for declaring significant QTLs. Randomization in a total of 1000 permutations with a *P* value threshold of 0.05 was used to verify LOD values. Hilum QTLs associated with quality and yield QTLs were identified as those that are linked closely enough to contain overlapping regions when QTLs were integrated and drawn in MapChart2.2 (Voorrips, 2002).

## Results

### Phenotypic Evaluation of the Two Parents

**FIGURE 1:**
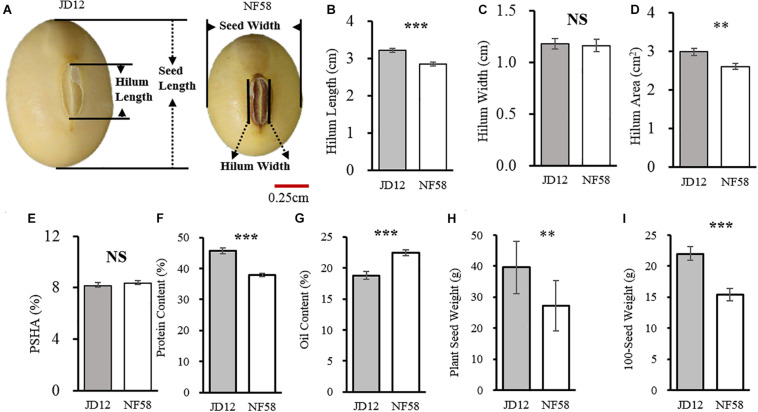
Comparison of hilum traits between soybean parent lines JD12 and NF58. **(A)** Photos of JD12 and NF58 seeds. **(B)** Seed hilum length (SHL). **(C)** Seed hilum width (SHW). **(D)** Seed hilum area (SHA). **(E)** Percentage of hilum area in seed projected area (PSHA). **(F)** Protein content (PC). **(G)** Oil content (OC). **(H)** Plant seed weight (PSW). **(I)** Hundred seed weight (100SW). Bars represent means ± SE from 15 replications. Asterisks indicate significant differences between JD12 and NF58 in the Student’s *t* test at the *P = 0.05, **P = 0.01, and ***P = 0.001 levels.

Before outlining the genetic basis of the traits tested herein, the two parents were first evaluated for phenotypic variation. Observed results from the field revealed that the two parents, JD12 and NF58, contrasted in hilum size, yield, and quality performance but did not significantly vary in HW and PSHA (Figure 1). JD12 had larger SHA values than NF58 (Figure 1D) due to longer SHL values (Figure 1B). However, that trend did not transfer to PSHA, which did not vary between the two parents (Figure 1E) because JD12 produced larger seeds than NF58, as indicated by a 42.54% higher 100SW for JD12 than for NF58 (Figure 1I). In addition, significant variation (*P* < 0.001) was also observed in PC (Figure 1F) and OC (Figure 1G), as well as in PSW (Figure 1H). Considering the importance of the hilum in seed development, variation in hilum size might contribute to variation in yield and quality traits between the two parents, which was further examined in the following analysis.

### Phenotypic Variation Among RILs

**FIGURE 2:**
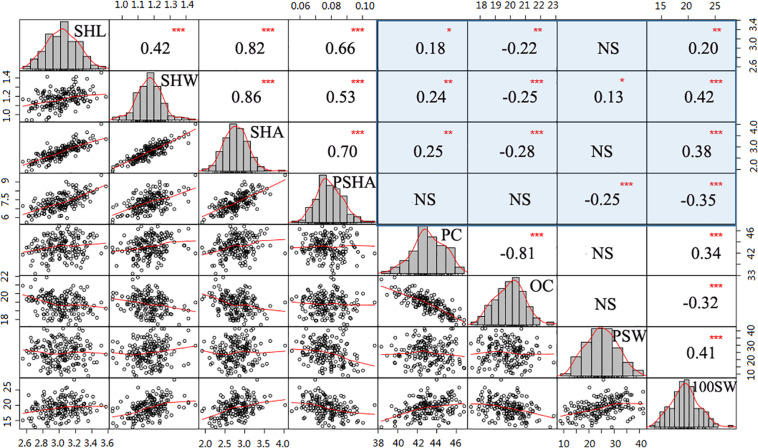
Correlation analysis among hilum, quality, and yield traits. Histograms with fitting curves of traits are graphed in the diagonal. Above the diagonal are correlation coefficients with significant levels. Below the diagonal are scatter plots with fitting curves. Red asterisks indicate significant correlations at the *P = 0.05, **P = 0.011, and ***P = 0.001 levels.

In order to identify QTLs for the eight tested traits, including four associated with seed hilum size, two with seed quality, and two with seed yield, the phenotypic variation of these traits was evaluated under field conditions from 2014 to 2016, with the results being summarized in Table 1. Significant phenotypic variation and extensive transgressive heritability among the observed 175 F_9_ soybean RILs was observed for each of the eight tested traits. The mean population value for each trait fell between the average parent values, while the maximum and minimum value fell beyond the extremes of the parent values. These results strongly indicate the presence of genetic variation between these two parents, which is necessary for follow QTL analysis. According to Kurtosis and Skewness values calculated over the 3 years of data, all eight tested fit into normal distributions (Table 1 and Figure 2). Meanwhile, broad-sense heritability (*h^2^_*b*_*) for the eight traits observed over 3 years of field experiments varied from 0.72 to 0.87 (Table 1), indicating that the phenotypic variation observed among RILs in this population was mainly derived from genetic variation. While neither SHW nor PSHA varied significantly between parents (Figures 1C,E), the yearly coefficient of variation for these traits varied from 5.88 to 7.54 and 10.25 to 12.07 for SHW and PSHA (Table 1), respectively, which strongly suggests that genetic variation underlies these traits that have not yet been fixed in this population. In short, these results demonstrate that significant variation was observed among the RILs observed here under field conditions, which is necessary to further identify QTLs for these tested traits in soybean.

**TABLE 1:** Phenotypic variation and genetic analysis of eight traits using 175 soybean recombinant inbred lines (RILs) observed under natural conditions.

Traits	Year	Parents		RILs								
		JD12	NF58	Max	Min	Mean	SD	CV/%	Skew	Kurt	*h^2^_*b*_*	*h^2^_*b(mean)*_*
SHL	2014	3.20	2.72	3.67	2.47	3.01	0.21	7.07	0.27	0.22	0.89	0.84
	2015	3.14	2.96	3.66	2.47	3.07	0.20	6.61	0.28	−0.17	0.84	
	2016	3.31	2.85	3.57	2.55	3.03	0.20	6.63	−0.39	0.07	0.84	
SHW	2014	1.18	1.16	1.31	0.99	1.13	0.07	6.41	−0.41	0.33	0.81	0.72
	2015	1.20	1.22	1.33	0.97	1.16	0.07	5.88	0.06	−0.17	0.77	
	2016	1.17	1.12	1.46	0.94	1.17	0.09	7.54	0.77	0.17	0.87	
SHA	2014	2.96	2.49	3.62	2.07	2.68	0.31	11.47	−0.08	0.50	0.88	0.79
	2015	2.95	2.83	3.65	2.09	2.80	0.28	10.13	−0.01	0.19	0.83	
	2016	3.04	2.50	3.66	1.92	2.78	0.32	11.37	0.54	0.26	0.88	
PSHA	2014	8.54	8.36	10.48	5.62	7.78	0.92	11.86	0.45	0.48	0.90	0.81
	2015	7.74	8.50	9.20	5.70	7.47	0.77	10.25	−0.34	−0.04	0.84	
	2016	8.23	8.11	10.85	5.46	7.77	0.94	12.07	0.06	0.30	0.88	
PC	2014	45.46	38.20	47.60	36.83	42.62	1.96	4.61	−0.46	−0.13	0.83	0.83
	2015	45.23	37.65	47.50	37.65	42.92	2.03	4.73	−0.28	−0.19	0.83	
	2016	46.44	37.87	47.71	37.88	43.22	2.04	4.72	−0.33	0.01	0.84	
OC	2014	19.05	22.19	22.54	16.07	19.60	1.19	6.09	−0.21	−0.02	0.84	0.87
	2015	19.12	22.23	23.24	17.09	19.99	1.18	5.92	−0.24	0.05	0.84	
	2016	18.13	22.79	22.81	17.49	20.02	1.13	5.62	0.29	−0.15	0.83	
100SW	2014	22.48	15.15	26.24	12.18	19.19	2.79	14.55	−0.15	0.22	0.84	0.87
	2015	21.44	16.12	27.98	13.16	19.35	2.87	14.84	0.19	0.26	0.82	
	2016	22.03	14.98	28.66	12.08	19.18	3.09	16.09	−0.23	−0.11	0.86	
PSW	2014	35.60	19.90	53.76	6.59	25.76	10.02	38.89	0.16	0.40	0.74	0.79
	2015	45.26	31.42	56.39	8.20	26.45	9.75	36.86	−0.57	0.04	0.70	
	2016	37.94	30.15	53.28	8.11	25.00	9.21	36.84	0.41	0.51	0.75	

*RILs, recombinant inbred lines. The eight observed traits included four hilum traits: SHL (seed hilum length, cm), SHW (seed hilum width, cm), SHA (seed hilum area, cm^2^), PSHA (percentage of seed hilum area in seed projected area, %), two quality traits PC (protein content, %) and OC (oil content, %); and two yield traits 100SW (100 seed weight, g) and PSW (plant seed weight, g).*

### Correlation Analysis of Tested Traits

To further determine whether hilum size can be a proxy trait in efforts to improve soybean seed yield or quality, correlation analysis was performed for the tested traits. In this analysis, values for the four hilum size traits were significantly correlated with those observed for most of the seed yield and quality traits (Figure 2). For instance, both SHL and SHW were correlated with PC, OC, and 100SW, with absoluter values ranging from 0.18 to 0.42 (*P* < 0.05). Moreover, relative higher correlation coefficients of SHW than that of SHL suggest that SHW might play more important roles than SHL in enhancing both seed yield and quality traits. Interestingly, SHA and PSHA played contrasting roles in improving seed yield and quality traits. For example, relatively large SHA values were associated with significantly enhanced (*P* < 0.001) 100SW (r = 0.38) and PC (r = 0.25) values but reduced OC (r = −0.28) values (*P* < 0.001). In contrast, PSHA had a significant negative impact (*P* < 0.001) on both PSW (r = −0.25) and 100SW (r = −0.35) but no effect on PC or OC values (*P* > 0.05). Taken together, these results suggest that incorporating hilum measurements into soybean selection efforts may contribute to programs seeking to breed high yielding or high-quality soybean cultivars.

### Construction of Genetic Linkage Maps

After filtering as described in *Materials and Methods*, 6407 SNPs remained for mapping. The constructed genetic linkage map covered 2621.6 cM with an average length of 133.7 cM per linkage group. Chromosome 13, at 271.0 cM, was the longest, and chromosome 16 was the shortest at 91.9 cM. The average number of SNPs on each linkage group was 320, with chromosome 18 having the most at 600 SNPs and chromosome 16 harboring the fewest at 143 SNPs. The average distance between adjacent SNPs was 0.42 cM, with these average distances varying between 0.22 cM on chromosome 16 and 0.64 cM on chromosome 16.

To check the accuracy of constructed linkage maps, the pubescence color trait controlled by the *T* gene (Toda et al., 2002) was also mapped. As expected, the *T* locus mapped to chromosome 6 between the 17,617,727 and 24,186,496 bp positions with an LOD value of 21.46. This result is consistent with the results of genome-wide association study (GWAS) displayed on www.soybase.org, which verifies that the linkage map constructed in this study was accurate and useful in further studies.

### Identification of QTLs for Hilum, Quality, and Yield Traits

In order to facilitate further MAS breeding, QTL analysis was performed for hilum, quality, and yield traits. This analysis returned a total of 53 significant QTLs for the tested traits, including 28 for hilum size traits, 12 for seed quality traits, and 13 for yield traits. For QTLs associated with hilum size, the phenotypic variation explained (PVE) values varied from 6.50 to 15.60%, and the LOD values ranged from 2.55 to 6.46 (Table 2). According to the genetic distances, these QTLs could be grouped into 11 loci; two of these, *qSH6.2* and *qSH8*, were stable loci over the 3 years of experimentation with PEV and LOD values of 7.30–15.60 and 2.88–6.43, respectively. For quality traits, the 12 significant QTLs detected in 3 years of field trials were localized to eight unique loci producing LOD and PVE values of 2.51–4.72 and 6.40–11.70, respectively (Table 3). However, most of these loci produced significant effects in only 1 or 2 years of trials. The exception was *qQ8*, with LOD and PVE values that peaked in the third year at 4.72 and 11.70, respectively. The combination of high LOD and PVE values being observed for all 3 years suggests that *qQ8* could be an important genetic determinant of protein and oil content in soybean. The 13 significant QTLs for yield traits were detected from seven unique loci explaining 6.70–16.10% of the phenotypic variation (Table 4). Among these loci associated with yield, only one locus, *qGY6.2*, with LOD values of 3.61–6.69 and explaining 9.10–16.10% of the phenotypic variation, could be detected in each of the 3 years of field trials. In summary, all of the results presented above suggest that, while all three of the tested traits were mainly impacted by minor QTLs that were sensitive to environmental conditions, the field trials still revealed four stable loci, namely, *qSH6.2*, *qSH8*, *qQ8*, and *qGY6.2*. These loci are good candidates for targeting in future soybean MAS breeding programs aiming to improve yield or quality.

**TABLE 2:** Putative quantitative trait loci (QTLs) for soybean seed hilum traits detected in a population of contrasting soybean parents and 175 recombinant inbred lines (RILs) reared under field conditions.

Integrated QTL	Separate QTL	Position	Chr	Locus/Interval	LOD	Add	PVE (%)	Year
*qSH2*	*qSHA2*	26.77	2	Gm02_5140506-6125494	3.03	0.09	7.70	2014
	*qSHW2*	26.77	2	Gm02_5140506-6125494	3.15	0.02	7.90	2014
*qSH4*	*qSHW4*	7.08	4	Gm04_717646-2052748	3.24	0.02	8.20	2015
*qSH6.1*	*qPSHA6.1*	53.50	6	Gm06_11700231-12028624	3.40	−0.28	8.50	2014
*qSH6.2*	*qPSHA6.2*	88.70	6	Gm06_17194761	4.79	−0.32	11.80	2016
	*qPSHA6.2*	90.70	6	Gm06_17194761-17617727	5.89	−0.36	14.40	2014
	*qPSHA6.2*	90.70	6	Gm06_17194761-17617727	2.88	−0.21	7.30	2015
	*qSHL6*	91.66	6	Gm06_17617727	3.04	−0.06	7.70	2014
	*qSHA6*	92.66	6	Gm06_17617727-24186496	5.97	−0.12	14.50	2014
	*qSHW6*	95.62	6	Gm06_24186496-41558653	6.43	−0.03	15.60	2014
	*qSHW6*	99.13	6	Gm06_41558653	3.63	−0.02	9.10	2015
*qSH8*	*qSHA8*	136.77	8	Gm08_44057851	3.57	0.10	9.00	2014
	*qPSHA8*	136.77	8	Gm08_44057851-44602360	3.10	0.28	7.80	2016
	*qSHL8*	137.77	8	Gm08_44057851-44602360	6.46	0.09	15.60	2014
	*qSHL8*	137.77	8	Gm08_44057851-44602360	4.75	0.07	11.80	2016
	*qSHL8*	144.95	8	Gm08_44826733-45270892	2.76	0.06	7.00	2015
*qSH10*	*qPSHA10*	89.10	10	Gm10_42920895	3.98	−0.30	9.90	2014
	*qSHW10*	98.97	10	Gm10_44648514-46136979	2.55	−0.03	6.50	2016
*qSH14*	*qSHW14*	104.77	14	Gm14_46973150-49265151	4.27	0.03	10.60	2014
*qSH16.1*	*qSHL16*	9.60	16	Gm16_1632510	2.58	0.05	6.60	2016
*qSH16.2*	*qSHW16.1*	74.89	16	Gm16_31154742-31956105	2.75	0.02	7.00	2014
*qSH16.3*	*qSHW16.2*	91.55	16	Gm16_33717504-37272971	2.71	0.02	6.90	2015
*qSH20*	*qPSHA20*	44.53	20	Gm20_27052809-32536925	3.59	−0.28	9.00	2014
	*qPSHA20*	44.53	20	Gm20_27052809-32536925	3.81	−0.24	9.50	2015
	*qSHA20*	44.64	20	Gm20_32536925	3.23	−0.09	8.10	2014
	*qSHA20*	44.64	20	Gm20_32536925	3.45	−0.08	8.70	2015
	*qSHL20*	44.64	20	Gm20_32536925	2.99	−0.06	7.60	2014
	*qSHL20*	44.64	20	Gm20_32536925	2.66	−0.05	6.80	2015

*Add values of > 0 and < 0 represent increasing effects of the QTLs derived from JD12 and NF58, respectively.*

**TABLE 3:** Putative quantitative trait loci (QTLs) for soybean quality traits detected in a population of contrasting soybean parents and 175 recombinant inbred lines (RILs) reared under field conditions.

Integrated QTL	Separate QTL	Position	Chr	Locus/Interval	LOD	Add	PVE (%)	Year
*qQ5*	*qPC5*	51.99	5	Gm05_32097530-32730716	2.54	0.45	6.50	2014
*qQ6*	*qOC6*	0.00	6	Gm06_1767951	2.60	−0.27	6.60	2016
*qQ7*	*qOC7*	45.56	7	Gm07_7098839-7607317	3.42	−0.31	8.60	2016
*qQ8*	*qOC8*	144.95	8	Gm08_44826733-45270892	2.70	−0.29	6.90	2014
	*qOC8*	144.95	8	Gm08_44826733	3.50	−0.34	8.80	2015
	*qOC8*	145.95	8	Gm08_44826733-45270892	4.72	−0.38	11.70	2016
*qQ10*	*qPC10*	91.10	10	Gm10_42920895-43696272	2.51	0.44	6.40	2014
*qQ11*	*qPC11*	19.57	11	Gm11_4216279	3.02	0.47	7.60	2014
	*qPC11*	19.57	11	Gm11_4216279	4.37	0.57	10.90	2015
*qQ12*	*qOC12*	105.52	12	Gm12_35953814-36347526	2.75	0.29	7.00	2016
*qQ13*	*qOC13*	241.11	13	Gm13_41203764	3.99	−0.33	10.00	2014
	*qOC13*	241.11	13	Gm13_41203764	2.75	−0.28	7.00	2015

*Add values of > 0 and < 0 represent increasing effects of the QTLs derived from JD12 and NF58, respectively.*

**TABLE 4:** Putative quantitative trait loci (QTLs) for soybean yield traits detected in a population of contrasting soybean parents and 175 recombinant inbred lines (RILs) reared under field conditions.

Integrated QTL	Separate QTL	Position	Chr	Locus/Interval	LOD	Add	PVE (%)	Year
*qGY6.1*	*q100SW6.1*	87.02	6	Gm06_16234959-17194761	3.50	0.82	8.80	2015
	*q100SW6.1*	92.66	6	Gm06_17617727-24186496	5.45	0.88	13.40	2016
	*qPSW6.1*	94.62	6	Gm06_24186496	5.94	2.86	14.50	2015
	*qPSW6.1*	94.62	6	Gm06_24186496	4.93	0.99	12.20	2016
*qGY6.2*	*qPSW6.2*	108.67	6	Gm06_44869374-46901447	5.78	3.31	14.10	2014
	*qPSW6.2*	111.66	6	Gm06_46901447-48098064	6.69	3.25	16.10	2015
	*q100SW6.3*	115.66	6	Gm06_46901447-48098064	3.61	0.76	9.10	2016
*qGY10*	*qPSW10*	98.97	10	Gm10_44648514-46136979	5.27	−2.97	12.90	2015
	*qPSW10*	100.97	10	Gm10_44648514-46136979	3.75	−2.86	9.40	2014
*qGY12*	*q100SW12*	125.95	12	Gm12_38306871	2.64	−0.61	6.70	2016
*qGY15*	*qPSW15*	64.64	15	Gm15_10944368-11421179	3.03	−2.37	7.70	2014
*qGY17*	*q100SW17*	109.86	17	Gm17_39691796	3.39	−0.84	8.50	2014
*qGY18*	*q100SW19*	97.78	19	Gm19_45525374	2.70	−0.62	6.90	2016

*Add values of > 0 and < 0 represent increasing effects of the QTLs derived from JD12 and NF58, respectively.*

### Overlapping Genetic Regions of Hilum, Quality and Yield Traits

**FIGURE 3:**
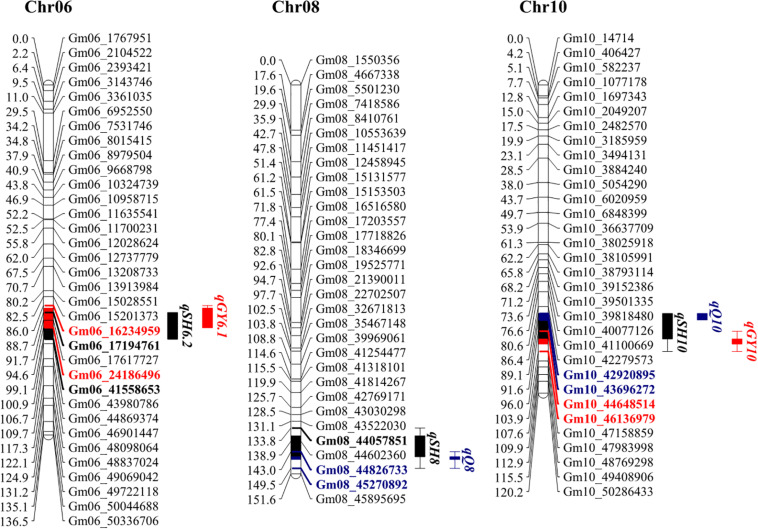
Genetic maps of loci associated with hilum size, seed quality, and soybean yield on chromosomes 6, 8, and 10. Colored and bold fonts represent loci for identified genes. Black, blue, and red blocks represent hilum, quality, and yield loci, respectively. Projected regions are highlighted in corresponding colors. Markers in different colors indicate corresponding markers on chromosomes and in projection regions.

One of the main objectives of this study is to identify genetic elements that affect all three categories of observed traits, hilum size, yield, and quality traits. Therefore, the confidence intervals for QTLs were projected on a genetic map. This revealed three overlapping regions located on Chr06, Chr08, and Chr10 (Figure 3). One of the two stable QTLs for hilum size, *qSH6.2*, colocated with *qGY6.1* on Chr06, and *qSHW6*, which contributed the highest LOD and PVE values to *qSH6.2* effects (Table 2), is also associated with *qGY6.1.* This colocalization coincided with the result that both PSW and 100SW were most highly correlated with SHW (Figure 2). Meanwhile, the other stable QTL for hilum size, *qSH8*, colocated with *qQ8* on Chr08, and *qSHL8*, which contributed the highest LOD and PVE values to *qSH8* (Table 2), was also closely aligned with *qQ8*. Another region of colocalization on Chr10 contained *qSH10*, *qQ10*, and qGY10. However, *qSH10*, *qQ10*, and *qGY10* were not stable QTLs across all 3 years of field trials. This region of colocated QTLs, therefore, should be further evaluated before including it in MAS breeding. At any rate, the overlapping of stable QTLs that was observed strongly suggests that SHW might be a good predictor of the seed yield, while SHL might be a good predictor of the seed quality, both of which could be considered in further soybean breeding efforts.

## Discussion

Soybean yield and quality are complex agronomic traits resulting from complex effects of many environmental and genetic factors (Li et al., 2008). In traditional breeding programs, successful breeding of high yielding cultivars with protein- or oil-rich seeds required many years of accumulated breeding experience. For example, in order to effectively select elite, high yielding soybean varieties, traditional breeding programs would rely on experienced breeders to comprehensively evaluate highly correlated traits, such as plant height, time to flowering time, time to maturity, and branch number, which have been identified over years of observation and which are now known to be physiologically associated with yield (Kato et al., 2018; Sun et al., 2019; Liu et al., 2020). Moreover, these accumulated experiences were mainly passed on orally, so new breeders needed many years of practice to fully comprehend breeding programs.

Other traits, such as seed oil and protein content, cannot be directly evaluated in the field, and known correlations with visible traits were previously non-existent. As a result, quality traits, such as protein and oil content, were rarely considered in traditional breeding operations, which increases the difficulty of breeding cultivars producing high quality seed.

Given this background of difficulties in breeding for yield and seed quality traits directly, breeders have incorporated the strategy of identifying stable and readily observable traits that are highly correlated with soybean yield and seed quality traits, especially new breeders. Although the seed hilum, which is easily evaluated and has proven to be critical for seed development and yield production (Hardham, 1976; Thorne, 1981), breeders have not until now considered it as proxy trait in efforts to improve soybean yield and quality because the genetic basis of seed hilum development and morphology were largely unknown.

In this study, the correlation of seed hilum size with soybean yield and quality traits was first determined under field conditions across three consecutive years of cultivation. Relatively higher heritability values (*h^2^_*b*_* = 0.72–0.90) for hilum traits were observed over these 3 years, which suggests that parameters of hilum morphology are stable traits with low sensitivity to environmental effects. Plus, most of the tested hilum size traits were significantly (*P* < 0.05) and highly correlated with both seed yield (|r| = 0.13–0.42) and quality traits (|r| = 0.18–0.28). Taken together, these results demonstrate that hilum size can be used as a simple correlated trait in efforts to breed higher yielding or improved quality varieties.

Numerous studies have been conducted with traits that are readily visible on soybean seeds, such as seed coat color, hilum color, and coat cracking, and which can have considerable impacts on commercial value (Oyoo et al., 2010; Guo and Qiu, 2013; Sonah et al., 2015; Cho et al., 2017; Saruta et al., 2019). Interestingly, some of these visual traits are also highly correlated with seed yield and/or quality traits. For example, seed coat cracking under low-temperature conditions can be significantly inhibited by the T gene responsible for pubescence color, along with the maturity genes, *E1* and *E5* (Yang et al., 2002), and the *T* and *E2* loci have also been associated with the severity of seed coat cracking induced by pod removal (Yang et al., 2002). In another work, hilum color was closely correlated with seed isoflavone abundance in a set of 17 contrasting soybean varieties (Barion et al., 2016). Hilum color has also been associated with seed size and yield in work where soybean varieties with large seeds and lighter colored hilums produced high seed yields in field conditions (Ladia et al., 2019). In addition, soybeans with brown hilums have been found on average to grow more vigorously and be tolerant of cold weather stress than soybeans with yellow hilums (Kurosaki et al., 2004), with the two hilum color associated loci, *Hilum color 2-g1* and *Hilum color 2-g2.1*, being localized to Chr06 and Chr08 (Sonah et al., 2015).

In this study, we identified 11 loci impacting seed hilum size, three of these, *qSH6.2*, *qSH8*, and *qSH10*, colocated with loci affecting seed yield and quality traits. Interestingly, based on the physical positions of flanking markers, *qSH6.2* mapped closely with the *T* locus (Hilum color 2-g1) and *qSH10* mapped closely with *E2.* Meanwhile, a recent report revealed that two Clark isolines with contrasted genotype at *T* locus displayed significant different in phenotype of hilum size (Zabala et al., 2020). Therefore, we hypothesize that *qSH6.2* and *qSH10* might be regulated by *T* and *E2*, respectively. Testing of this hypothesis fell beyond the scope of this work and will require further investigation. In contrast to *qSH6.2* and *qSH10*, *qSH8*, which was mapped to Chr08:44057851–45270892, did not colocalize with any well-known gene locus, suggesting that this might be a novel avenue to explore in attempts to breed high yielding or high quality soybean varieties.

In summary, we provide a preliminary description here of potential roles for genetic elements associated with seed hilum size in breeding programs aimed at improving soybean yield and seed quality. The observations reported herein identified three genetic loci that might be valuable in MAS breeding efforts.

## Data Availability Statement

The raw data supporting the conclusions of this article will be made available by the authors, without undue reservation.

## Author Contributions

QZ, XS, YY, and MZ designed the experiments and analyzed the data. QZ, YY, CY, CL, and YF carried out the experiments. XS, QZ, and LY constructed and genotyped the RILs. HL, QZ, and YY wrote the manuscript. All authors contributed to the article and approved the submitted version.

## Conflict of Interest

The authors declare that the research was conducted in the absence of any commercial or financial relationships that could be construed as a potential conflict of interest.
