# Supplementary figures and images for: Characterization of the Common Genetic Basis Underlying Seed Hilum Size, Yield, and Quality Traits in Soybean
Source: Front Plant Sci. 2021 Feb 25;12:610214. doi: 10.3389/fpls.2021.610214 (PMC7947287; doi:10.3389/fpls.2021.610214)

## Slide 1
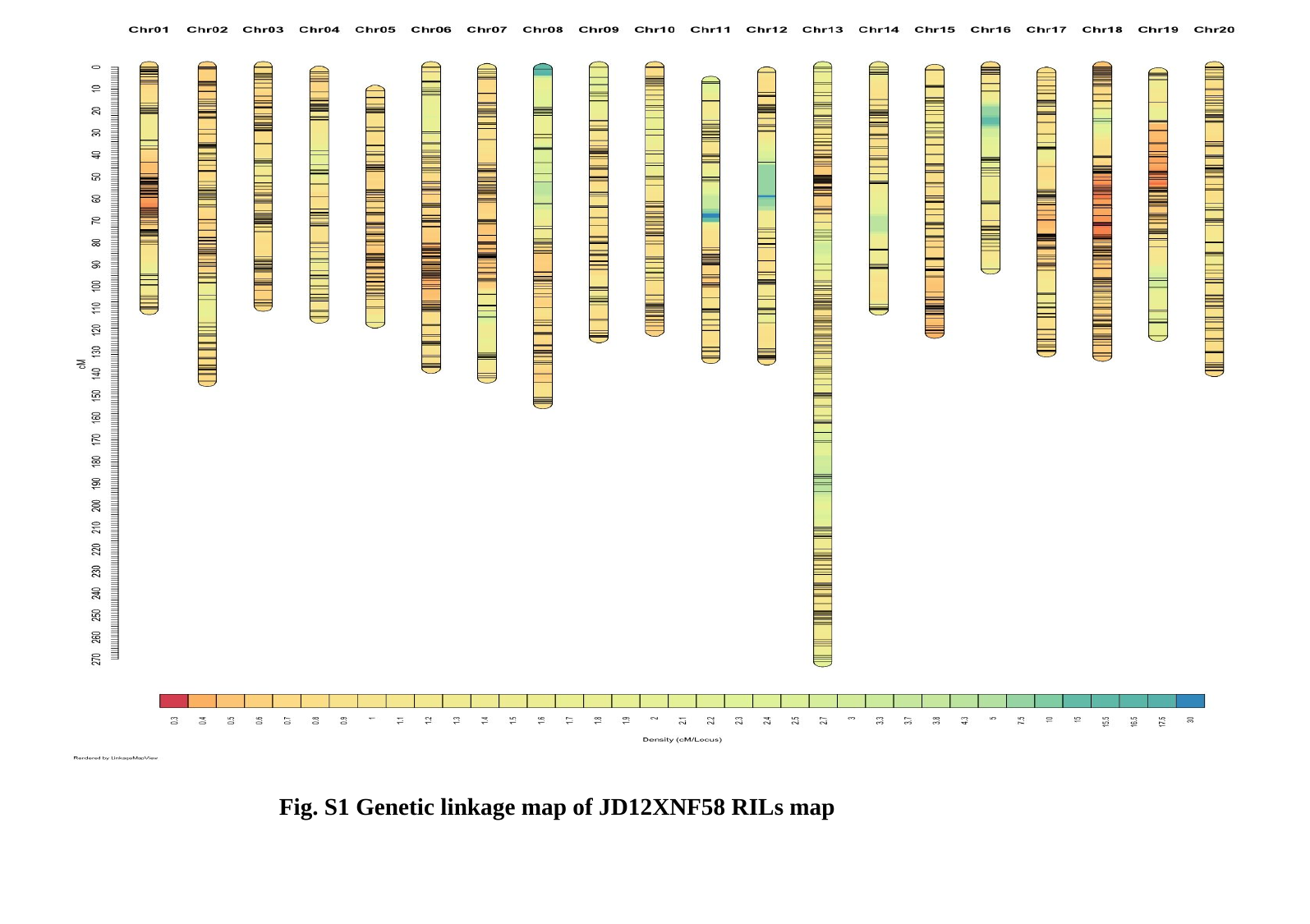

Fig. S1 Genetic linkage map of JD12XNF58 RILs map

Supplement: Supplementary file 1 [file Presentation_1.PPTX]
